# 512. Efficacy of Gepotidacin versus Nitrofurantoin in a Nitrofurantoin Not Susceptible Population: A Pooled Analysis of the EAGLE-2 and EAGLE-3 Randomized Controlled Trials in Uncomplicated Urinary Tract Infection

**DOI:** 10.1093/ofid/ofae631.164

**Published:** 2025-01-29

**Authors:** Jeremy Dennison, Amanda Sheets, Sarah Watts, Nicole E Scangarella-Oman, Deborah Butler, John Breton, Salim Janmohamed

**Affiliations:** GSK, Brentford, UK, Brentford, England, United Kingdom; GSK, Collegeville, PA, USA, Collegeville, Pennsylvania; GSK, Brentford, England, United Kingdom; GlaxoSmithKline plc., Collegeville, Pennsylvania; GSK, Brentford, England, United Kingdom; GSK, Brentford, England, United Kingdom; GSK, Brentford, UK, Brentford, England, United Kingdom

## Abstract

**Background:**

Two Phase 3 trials (EAGLE-2 [NCT04020341]/EAGLE-3 [NCT04187144]) showed that gepotidacin, a first-in-class triazaacenaphthylene antibacterial, was non-inferior to nitrofurantoin (NTF) in treating uncomplicated urinary tract infection (uUTI) caused by NTF-susceptible (NTF-S) qualifying uropathogens (≥ 10^5^ CFU/mL).^1^ This study evaluates gepotidacin efficacy in the pooled EAGLE-2/3 microbiological intent-to-treat (micro-ITT) NTF not susceptible (NTF-NS) population.

**Figure:**
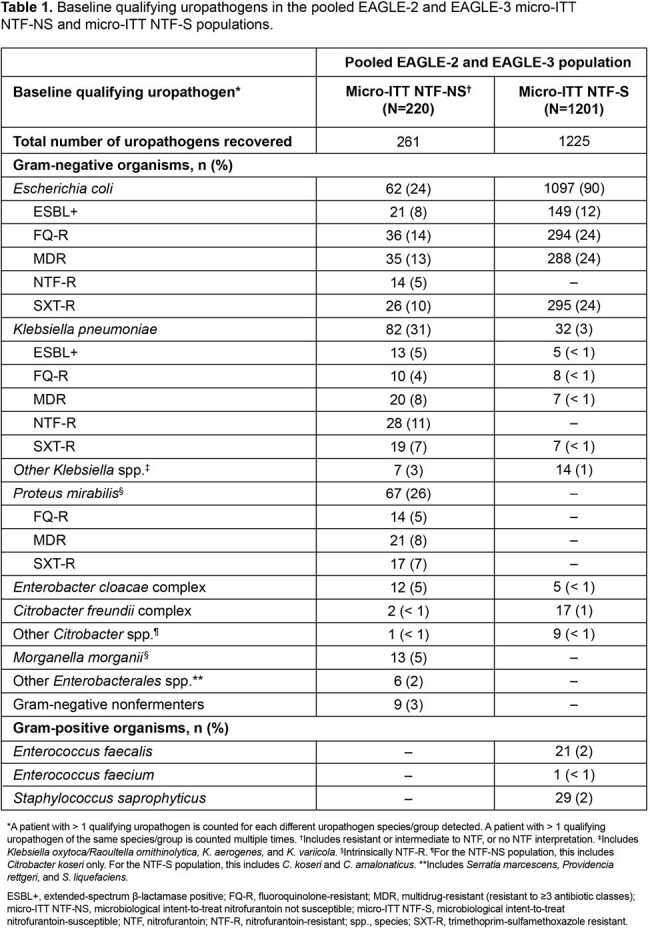

**Methods:**

In EAGLE-2/3, female patients aged ≥ 12 years with ≥ 2 uUTI symptoms received oral gepotidacin (1500mg) or NTF (100mg) twice-daily for 5 days.^1^ In this study, therapeutic, clinical and microbiological response (defined in tables) at test-of-cure (Day 10–13) were assessed in the pooled EAGLE-2/3 micro-ITT NTF-NS and NTF-S populations and across patient subgroups.

**Figure:**
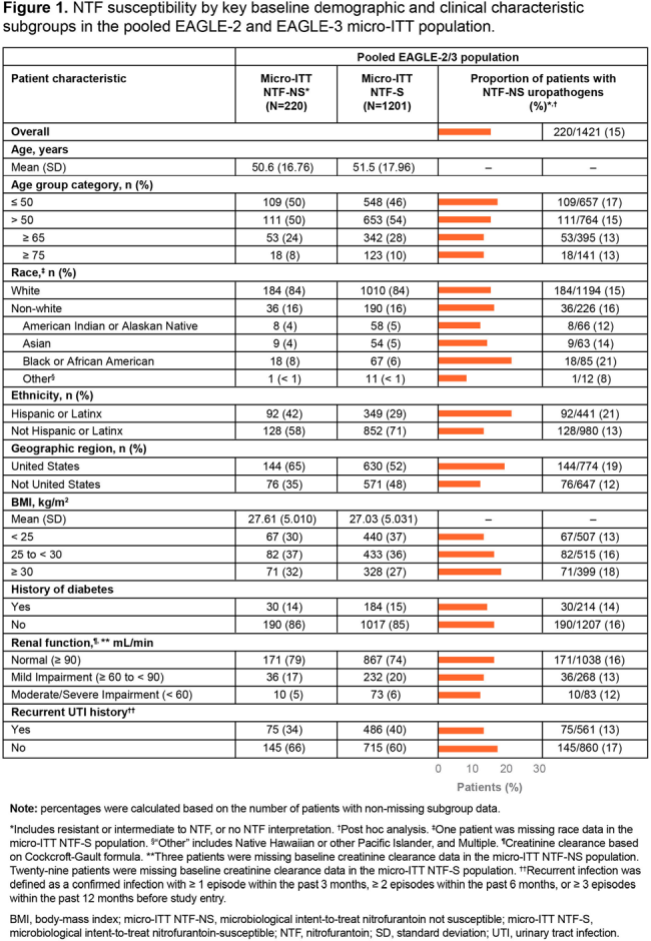

**Results:**

Of the 1421 micro-ITT patients, 220 (15%) had NTF-NS uropathogens and 1201 (85%) had NTF-S uropathogens. The most common baseline uropathogens were *Escherichia coli* (90%) in the NTF-S population, and *Klebsiella pneumoniae* (31%), *Proteus mirabilis* (26%), and *E. coli* (24%) in the NTF-NS population (**Table 1**). Across key subgroups, the proportion of patients with NTF-NS uropathogens was broadly similar to the overall population (except Hispanic/Latinx, Black race and US subgroups which were numerically higher; **Figure 1**). For efficacy outcomes, treatment differences were higher in the NTF-NS vs the NTF-S population, and, as expected, favored gepotidacin vs NTF (**Table 2**). Treatment differences in therapeutic success were numerically higher across key subgroups in the NTF-NS vs the NTF-S population (**Figure 2**).

**Figure:**
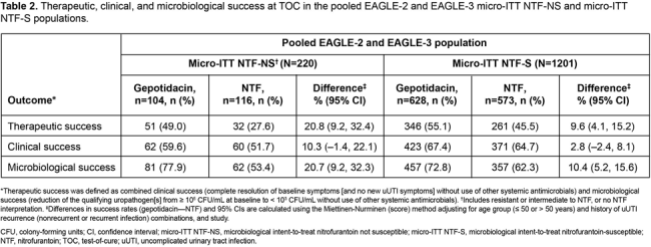

**Conclusion:**

Gepotidacin was efficacious in both populations, and across subgroups, with a higher therapeutic treatment difference in the NTF-NS vs the NTF-S population. Gepotidacin could offer benefit in patients with uUTI, including those with NTF-NS uropathogens.

1. Wagenlehner F, et al. *Lancet* 2024;403:741–55.

**Funding:** EAGLE-2 was funded in part by GSK and in part with Federal funds from the US Office of the Assistant Secretary for Preparedness and Response, Biomedical Advanced Research and Development Authority (HHSO100201300011C). EAGLE-3 was funded by GSK.

**Figure:**
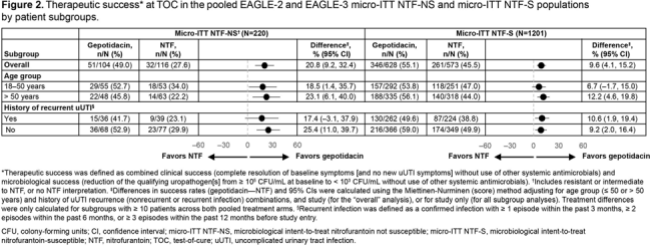

**Disclosures:**

**Jeremy Dennison, MD PhD**, GSK: Employee|GSK: Stocks/Bonds (Public Company) **Amanda Sheets, PhD**, GSK: Employee|GSK: Stocks/Bonds (Public Company) **Sarah Watts, MSc**, GSK: Employee|GSK: Stocks/Bonds (Public Company) **Nicole E. Scangarella-Oman, MS**, GSK: Employee|GSK: Stocks/Bonds (Public Company) **Deborah Butler, PharmD**, GSK: Employee|GSK: Stocks/Bonds (Public Company) **John Breton, MCM**, GSK: Employee|GSK: Stocks/Bonds (Public Company) **Salim Janmohamed, MD**, GSK: Employee|GSK: Stocks/Bonds (Public Company)

